# Skipping breakfast is detrimental for primary school children: cross-sectional analysis of determinants for targeted prevention

**DOI:** 10.1186/s12889-017-4169-z

**Published:** 2017-03-14

**Authors:** Dorothea Kesztyüs, Meike Traub, Romy Lauer, Tibor Kesztyüs, Jürgen Michael Steinacker

**Affiliations:** 1 Division of Sport and Rehabilitation Medicine, Ulm University Medical Center, 89075 Ulm, Germany; 2 Institute of General Medicine, Ulm University, Helmholtzstraße 20, D-89081 Ulm, Germany; 3 Department of Computer Science, Ulm University of Applied Sciences, 89081 Ulm, Germany

**Keywords:** Child, Food habits, Breakfast, Health promotion, Abdominal obesity, etiology, prevention & control

## Abstract

**Background:**

Skipping breakfast was found to be associated with abdominal obesity in primary school children. The aim of this research was to examine factors associated with skipping breakfast in primary school children in order to develop targeted preventive measures.

**Methods:**

Baseline data assessment (2010) of a cluster-randomized controlled trial for the evaluation of a school-based health promotion program in primary school children in the state of Baden-Württemberg, Germany. Anthropometric measures of 1,943 primary school children aged 7.1 ± 0.6 years (51.2% boys) were conducted according to ISAK-standards (International Standard for Anthropometric Assessment) by trained staff. Further information on the health and living conditions of the children and their parents were assessed in parental questionnaires. Generalized linear mixed regression analysis was calculated to define correlates for skipping breakfast in terms of odds ratios (OR) and 95% confidence intervals (CI).

**Results:**

According to the final regression models, significant correlates of skipping breakfast can be divided into modifiable behavioral components (high consumption of soft drinks (OR 2.49, 95% CI 1.81; 3.43), screen media (OR 2.48, 95% CI 1.77; 3.46) and high levels of physical activity (OR 0.64, 95% CI 0.44; 0.93)) on the one hand, and more or less static socio-economic factors (migration background (OR 2.81, 95% CI 2.02; 3.91), single parenting (OR 2.13, 95% CI 1.34; 3.40), and high family education level (OR 0.42, 95% CI 0.28; 0.64)) on the other hand, and finally individual factors (female gender (OR 1.43, 95% CI 1.03; 1.99) and having a percentage of body fat at or above the 95th percentile (OR 1.47, 95% CI 1.00; 2.17)).

**Conclusion:**

Targeted prevention should aim at health-related behaviors accompanying the habit of skipping breakfast. Focusing on vulnerable groups, characterized by not so easily modifiable socio-economic as well as individual factors, may improve results. Interventions should synergistically promote children’s health and involve their parents in order to be successful. To reach all children and to avoid skipping breakfast, schools should offer regular breakfast at the start of a school day. Policy makers should support healthy eating habits at all times.

## Background

There is considerable evidence from systematic reviews for children and adolescents that eating breakfast is associated with a reduced risk of becoming overweight or obese and a reduction in body mass index (BMI) [1, 2]. For example, Swiss children regularly consuming breakfast showed better motor functional skills and were less overweight [3]. For schoolchildren, breakfast plays a positive role in maintaining cognitive function during the morning [4]. Furthermore, English children who regularly consumed breakfast had a more favorable type 2 diabetes risk profile [5] and Greek schoolchildren showed an inverse association of breakfast consumption with HOMA-IR (homeostasis model assessment of insulin resistance index) [6]. In obese children and adolescents, skipping breakfast is correlated with higher levels of blood glucose, triglycerides and very low density lipoprotein cholesterol [7]. Finally, in Canadian children and adolescents, breakfast consumption is positively associated with nutrient adequacy [8].

In a recent study, we identified skipping breakfast as one of the modifiable influencing factors for developing abdominal obesity in primary schoolchildren [9]. This finding is supported by a study of Alexander and colleagues who were able to demonstrate that higher visceral adiposity was associated with skipping breakfast in overweight Latino youth [10]. Abdominal obesity is more and more recognized as the most risky kind of obesity, as it is strongly associated with the majority of non-communicable diseases (NCD), the world’s number one killer [11, 12]. Furthermore, a remarkable number of persons who are normal weight according to BMI definition, are abdominally obese and e.g. for subjects with coronary artery disease, those who are normal weight but abdominally obese carry the highest risk of mortality [13]. Concerning children, a meta-analysis revealed that BMI fails to identify excess adiposity in over a quarter of the affected children [14]. A longitudinal study comparing BMI and waist circumference in children concludes “Children appear to be getting fatter and the additional adiposity is being stored centrally which is not detected by BMI” [15]. Those children with abdominal obesity already have a lower health related quality of life, more days of absence at school and more visits to a physician [16].

According to the literature, the frequency of breakfast intake decreases with age in children and adolescents [1, 8, 17]. Systematic reviews report associations of skipping breakfast in youth with lower socioeconomic status, lack of physical activity, frequent use of screen media, higher energy intake, unhealthy eating habits and other unhealthy lifestyle factors such as smoking and alcohol use [1, 17]. All in all, girls were more likely to skip breakfast than boys [1, 17].

Our study was embedded in the outcome evaluation of the health promotion program “Join the Healthy Boat”. This program includes health promotion in the curriculum of grades one to four at primary school and combines behavioral and environmental components. Children are empowered to make healthy choices in terms of physical activity, consumption of sugar sweetened beverages, and use of screen media. The aim of the present study is to look at factors that are associated with skipping breakfast in schoolboys and -girls in order to improve preventive and health promoting measures and to help respective multicomponent interventions to reach their goal of promoting healthy weight in children.

## Methods

The health promotion program “Join the Healthy Boat” was based on the successful “URMEL ICE” project (Ulm Research on Metabolism, Exercise and Lifestyle Intervention in Children), to the author’s knowledge the only school-based prevention program in Germany with proven cost-effectiveness [18].

### Study design

The present study was embedded in the baseline-measurements of the outcome evaluation of the school-based health promotion program “Join the Healthy Boat” in the state of Baden-Württemberg, Germany, in the year 2010. The “Baden-Württemberg Study” followed a cluster-randomized design with a waitlist control group. A more detailed description of the study can be found elsewhere [19].

### Participants and data

All children in classes of teachers who agreed to participate in the outcome evaluation were eligible (3,159 pupils). Parents of 1,968 first and second graders in 84 schools (5.4 – 9.9 years old), 62% of all eligible children, gave their consent for participation in 2010. Response rate for parental questionnaires was 87% at baseline. Data from direct measurements at schools or from parental questionnaires were available for 1,943 children at baseline. Parents gave information on their health behavior and their socioeconomic background, as well as on the health and lifestyle characteristics of their children.

### Demographics

The parental education level was assessed and determined according to the CASMIN (Comparative Analysis of Social Mobility in Industrial Nations) educational classification [20]. CASMIN classifies tertiary level as higher educational levels (e.g. academically-oriented university education), while secondary level includes a range from intermediate vocational qualification to full maturity certificates (equals 12–13 years of school in Germany), and primary level comprises inadequately completed general education to basic vocational qualification. Family education level was defined as the highest level of two parents or the level of a single parent who mainly cares for the child. For analyses, family education level was dichotomized into tertiary versus intermediate and elementary level. Household income was assessed in several categories according to the German KiGGS survey (German Health Interview and Examination Survey for Children and Adolescents) [21] and dichotomized for analyses at a threshold of €1,750. A migration background was assumed if at least one parent was born abroad or at least one parent had mainly spoken a foreign language during the child’s first years of life.

### Health and lifestyle characteristics

Questions about children’s behavior were taken from the validated questionnaires of the German KiGGS survey [22]. Answers were offered on a 5-point Likert scale for frequency of consuming soft drinks, frequency of playing outside (nearly every day, 3–5 times a week, 1–2 times a week, less than 1 time a week, never), and time spent with screen media (never, about 30 min/day, about 1–2 h/day, about 3–4 h/day, more than 4 h/day). Variables were dichotomized for analyses (soft drinks > 1/week, playing outside > 60 min/day, screen media > 1 h/day). Information on the frequency of participation in club sports were retrieved in an open question and results were dichotomized for analyses (> 1/week). Parents gave information on the frequency of breakfast before school for their children on a 4-point scale, the results were subsequently dichotomized for analyses (never, rarely vs. often, always). Furthermore, parents stated how many days a week their children were physically active on a moderate to vigorous level (starting to sweat and/or get out of breath) for at least 60 min a day, as recommended by the World Health Organization (WHO) [23]. Results were dichotomized for analyses at the median (physically active ≥ 4 days/week ≥ 60 min/day). Lastly, parents were asked whether they were smokers, and they rated their health awareness on a 4-point scale, the latter variable being then dichotomized for analyses (not at all, little vs. strong, very strong).

### Anthropometric measurements

Trained staff conducted the anthropometric measurements of the children according to ISAK-standards [24]. The children’s height was measured to the nearest 0.1 cm (Stadiometer, Seca®, Germany), and body weight to the nearest 0.1 kg using calibrated and balanced portable digital scales (Seca®, Germany). Waist circumference (WC) was measured midway between ileac crest and lower costal arch to the nearest 0.1 cm using a flexible metal tape (Lufkin Industries Inc., Texas, USA). The children’s BMI was calculated as weight divided by height squared [kg/m^2^]. Excess weight and obesity were defined at or above the 90^th^ and 97^th^ age- and gender-specific BMI percentiles according to German reference data [25]. Waist- to-height ratio (WHtR) was calculated as the ratio of WC and height in centimeters, participants with a WHtR ≥ 0.5 were then categorized as abdominally obese [26]. Because there was some doubt the WHtR threshold of 0.5 was sensitive enough, a lower threshold (0.47 for girls and 0.48 for boys) as proposed by Nambiar et al. [27], correlating with the 95^th^ percentile for %body fat, was added.

Parental BMI was calculated with self-reported weight and height data from the questionnaires, and categorized as overweight (BMI ≥ 25.0) and obese (BMI ≥ 30.0), according to the international classification of the WHO [28]. Parental WHtR was calculated as self-reported WC divided by height in centimeters and abdominal obesity was defined as WHtR ≥ 0.5 [29].

### Missing data

Missing data are a frequently occurring problem in observational studies, possibly leading to biased results [30]. Therefore, potential significant baseline differences between cases with and without missing values for the full regression model were examined.

### Statistical analysis

Differences between boys and girls, as well as between participants with and without missing values, were tested for their statistical significance. Depending on scale level and distribution of the data, the Mann–Whitney-*U* test or *t*-test for continuous data and Fisher’s exact test for categorical data were applied. The significance level was set at α = 0.05 for two-sided tests. These analyses were carried out using the statistical software packages IBM SPSS Release 21.0 for Windows (SPSSInc, Chicago, IL, USA).

To identify a possible clustering effect of data in schools, the intraclass correlation coefficient (ICC_Logit_) for a generalized linear mixed model with binary outcome was calculated according to Eldridge et al. [31]. Depending on the magnitude of the ICC_Logit_, the appropriate regression analysis, a logistic or a generalized linear mixed model, was conducted subsequently, considering the variables described above. A closer examination of decisive factors for target groups and target behaviors was realized in two sub-models. These analyses were performed with the statistical software R Release 3.2.5 for Windows (http://cran.r-project.org/).

## Results

### Baseline characteristics

Primary school children who took part in this research had a mean age of 7.1 ± 0.6 years, 51.2% of them were boys. Table 1 shows the baseline characteristics of the participants. Significant differences between boys and girls occurred in anthropometric variables where boys had a slightly higher waist circumference but were less frequently at or above the 95^th^ percentile for %body fat. They also had a different distribution of WHtR with a lower variance and range than girls. Boys more often played outside and reached higher levels of physical activity. Girls more often skipped breakfast than boys.

**Table 1:** Baseline characteristics of participants in the Baden-Württemberg Study (2010)

	Missing	Boys	Girls	Total
	Values	(*n* = 995)	(*n* = 948)	(*n* = 1,943)
Child characteristics				
Age, years [m (sd)]		7.09 (0.64)	7.06 (0.63)	7.08 (0.64)
Migration background, *n* (%)	297	255 (30.9)	270 (32.9)	525 (31.9)
BMIPERC, [m (sd)]	50	48.78 (27.87)	49.14 (27.92)	48.96 (27.89)
Overweight, *n* (%)	50	54 (5.6)	54 (5.9)	108 (5.7)
Obesity, *n* (%)	50	49 (5.1)	34 (3.7)	83 (4.4)
Waist circumference, [m (sd)]	55	55.98 (5.83)***^a^	55.16 (5.90)	55.58 (5.88)
WHtR, [m (sd)]	55	0.45 (0.04)*	0.45 (0.04)	0.45 (0.04)
Abdominal obesity, *n* (%)	55	73 (7.5)	85 (9.2)	158 (8.4)
≥ 95^th^ percentile for %body fat, *n* (%)	55	128 (13.2)***^a^	211 (22.9)	339 (18.0)
Parental characteristics				
Single parent, *n* (%)	265	82 (9.7)	95 (11.4)	177 (10.5)
Tertiary family educational level, *n* (%)	215	262 (31.8)	261 (32.5)	523 (32.1)
Household income ≤ €1,750, *n* (%)	452	101 (13.4)	106 (14.4)	207 (13.9)
Overweight/obesity (mother or father), *n* (%)	332	532 (65.0)	542 (68.3)	1074 (66.7)
Abdominal obesity (mother or father), *n* (%)	974	405 (82.7)	407 (85.0)	812 (83.8)
Smoking (mother or father), *n* (%)	270	309 (36.9)	319 (38.2)	628 (37.5)
Health awareness (mother or father), *n* (%)	343	541 (67.4)	539 (67.6)	1080 (67.5)
Health and lifestyle characteristics				
Skipping breakfast, *n* (%)	236	89 (10.4)**	134 (15.8)	223 (13.1)
Soft drinks > 1/week, *n* (%)	241	219 (25.6)	197 (23.3)	416 (24.4)
Playing outside > 60 min/day, *n* (%)	296	615 (73.8)***	515 (63.3)	1130 (68.6)
Physically active ≥ 4 days/week ≥ 60 min/day, *n* (%)	320	260 (31.7)***	177 (22.1)	437 (26.9)
Club sports > 1/week, *n* (%)	662	338 (52.0)	338 (53.6)	676 (52.8)
Screen media > 1 h/day, *n* (%)	250	173 (20.4)	146 (17.3)	319 (18.8)

*NOTE. m* mean, *sd* standard deviation, *BMI* body mass index, *BMIPERC* BMI percentiles, *WHtR* waist-to-height ratio

^a^Mann–Whitney-*U* test, *p < 0.05, **p < 0.01, ***p < 0.001

All variables shown in Table 1 were considered for their potential association with the outcome variable in the regression analyses.

### Regression analysis for correlates of skipping breakfast

Unadjusted, crude odds ratios (OR) for skipping breakfast for all variables in the subsequent generalized linear mixed regression models are illustrated in Table 2.

**Table 2:** Crude OR for independent variables taken into account in the generalized linear mixed regression model for skipping breakfast

	Missing	Crude	
	Values	OR	95% CI
Child characteristics			
Female gender	236	1.62	(1.22; 2.16)
Age > 7 years	281	1.04	(0.78; 1.37)
Migration background	303	3.60	(2.67; 4.84)
Overweight	471	2.02	(1.18; 3.43)
Obesity	286	1.94	(1.03; 3.66)
Abdominal obesity	290	2.51	(1.63; 3.88)
≥ 95^th^ percentile for %body fat	290	2.20	(1.58; 3.07)
Parental characteristics			
Single parent	271	2.45	(1.68; 3.58)
Tertiary family education level	321	0.34	(0.22; 0.50)
Household income ≤ €1,750	457	3.48	(2.46; 4.94)
Overweight/obesity (mother or father)	338	1.89	(1.33; 2.67)
Abdominal obesity (mother or father)	978	1.16	(0.66; 2.03)
Smoking (mother or father)	276	2.60	(1.94; 3.49)
Health awareness (mother or father)	349	0.70	(0.52; 0.96)
Health and lifestyle characteristics			
Soft drinks > 1/week	244	2.78	(2.07; 3.73)
Playing outside > 60 min/day	301	0.67	(0.50; 0.90)
Physically active ≥ 4 days/week ≥ 60 min/day	326	0.62	(0.43; 0.90)
Club sports > 1/week	667	1.03	(0.72; 1.48)
Screen media > 1 h/day	256	2.97	(2.18; 4.04)

*NOTE. N* = 1,943. *OR* odds ratio, *CI* confidence interval

In the bivariate analysis, female gender, migration background, variables of overweight and obesity, single parenthood, lower household income, parental overweight and obesity, parental smoking, one or more soft drinks per week, and screen media use exceeding 1 hour per day had higher odds for skipping breakfast. On the other hand, tertiary family education level, parental health awareness, playing outside more than 60 min per day, and physical activity of more than 60 minutes on 4 days and more per week showed lower odds for skipping breakfast. The ICC_Logit_ of skipping breakfast was 0.045, indicating that 4.5% of the total variance is due to clustering of data in schools. This would lead to differences in the ORs between a logistic regression model and a generalized linear mixed model. Therefore, the model presented here is the generalized linear mixed model for binary outcomes, which accounts for the clustering of data in schools. Table 3 shows both the results of the full model and of sub-models for groups and behaviors for targeted preventive measures.

**Table 3:** Generalized linear mixed models for skipping breakfast, full and sub-models for target groups and behaviors

	Full model		Sub-model target groups		Sub-model target behaviors	
	(*n* = 1,441)		(*n* = 1,515)		(*n* = 1,612)	
	OR	95% CI	OR	95% CI	OR	95% CI
Migration background	2.39***	(1.68; 3.40)	2.81***	(2.02; 3.91)		
Tertiary family education level	0.55**	(0.35; 0.85)	0.42***	(0.28; 0.64)		
Single parent	2.17**	(1.33; 3,54)	2.13**	(1.34; 3.40)		
Female gender	1.53*	(1.07; 2.18)	1.43*	(1.03; 1.99)		
≥95^th^ percentile for %body fat	1.51^†^	(0.99; 2.24)	1.47^†^	(1.00; 2.17)		
Soft drinks > 1/week	2.41***	(1.70; 3.44)			2.49***	(1.81; 3.43)
Physically active ≥ 4 days/week ≥ 60 min/day	0.71	(0.47; 1.07)			0.64*	(0.44; 0.93)
Screen media > 1 h/day	1.91***	(1.31; 2.79)			2.48***	(1.77; 3.46)

*NOTE. OR* odds ratio, *CI* confidence interval

†*p* < .10; *p < .05; **p<. 010; ***p < .001

A higher level of physical activity and tertiary family education level showed lower odds for skipping breakfast. On the other hand, migration background, single parenthood, female gender, percentage of body fat at or above the 95^th^ percentile, and frequent soft drink and screen media consumption had higher odds.

No significances were lost after dividing the full model into two separate sub-models for target groups and target behaviors. ORs in the sub-models were more pronounced than in the full model.

Further sub-models were calculated for boys and girls, they are depicted in Table 4.

**Table 4:** Generalized linear mixed models for skipping breakfast, sub-models for boys and girls

	Sub-model girls		Sub-model boys	
	(*n* = 767)		(*n* = 758)	
	OR	95% CI	OR	95% CI
Migration background	2.51***	(1.60; 3.93)	3.63***	(2.10; 6.28)
Tertiary family education level			0.36**	(0.17; 0.74)
Single parent	2.95***	(1.64; 5.31)		
≥95^th^ percentile for %body fat	1.56^†^	(0.97; 2.52)		
Soft drinks > 1/week	2.12**	(1.33; 3.38)	2.88***	(1.68; 4.94)
Physically active ≥ 4 days/week ≥ 60 min/day			0.55^†^	(0.29; 1.04)
Screen media > 1 h/day	2.62***	(1.60; 4.31)		

*NOTE. OR* odds ratio, *CI* confidence interval

†*p* < .10; *p < .05; **p<. 010; ***p < .001

The sub-model for girls showed higher odds for migration background, single parenthood, percentage of body fat at or above the 95^th^ percentile, and frequent soft drink and screen media consumption. In the sub-model for boys lower odds were found for a higher level of physical activity and tertiary family education level.

### Missing data

Participating children with missing data, significantly, more often had a migration background, lower results in the 6 min run test, and they differed in all anthropometric measurements from the ones with complete data. Participants with missing data had significantly higher BMI percentiles, more often were overweight and obese, and more often had a higher waist circumference and a higher WHtR. They also were more often abdominally obese and more often had a percentage of body fat at or above the 95^th^ percentile than their counterparts. Finally, their parents were significantly more often single parents, less frequently had a tertiary educational level and more often a household income at or below €1,750.

## Discussion

The study shows that migration background, living with a single parent, female gender, having a percentage of body fat at or above the 95^th^ percentile, the consumption of soft drinks and high levels of screen media use are positively correlated with children skipping breakfast. Reaching high levels of physical activity and tertiary family education level are negatively correlated with skipping breakfast. Accordingly, interventions that influence the reported target behaviors as well as those that are tailored to the identified target groups are necessary. Behavioral changes can be addressed in all kinds of interventions at different levels. Differences between boys and girls should be taken into account according to the gender distribution in the respective target group.

First, parents should be informed about the importance of a healthy lifestyle and health-conscious behavior, such as responsible media consumption, sufficient physical activity, little or no soft drink consumption and most of all the importance of a regular breakfast. Furthermore, parents should be supported in their essential function as a role model for their children and demonstrate healthy breakfast habits. Therefore, interventions should synergistically promote children’s health and involve their parents in order to be successful [32]. At an organizational level, teachers could inform parents about the need for regular and healthy breakfast for children at parents’ evenings because there are positive effects of breakfast frequency and quality for both parents and children [33].

Some of the identified family-related factors for skipping breakfast are non- or hardly modifiable, like migration background, family education level and single parenthood. Despite these difficult socioeconomic circumstances (e.g. poor housing, living and working conditions, worries, uncertainty) in which parents have to bring up their children, many of them may be aware of the importance of a healthy lifestyle but lack the necessary resources to implement it. The most promising way to reach children from families with these traits runs via settings like schools or kindergarten. The latter should offer regular breakfast at the start of a school or kindergarten day. In this way, all children are reached and skipping breakfast can be avoided. When developing measures for targeted prevention, there should be a special focus on the needs of the target groups identified in this study. Families in difficult socioeconomic circumstances need to be supported and provided with financial assistance for the payment of breakfast and/or other healthy meals at school. Thus, policy makers and intervention developers have to note that there are low-threshold and easily accessible opportunities required for reaching deprived target groups. Not least, policy makers should support healthy eating habits in schools and kindergartens at all times.

### Relevance of breakfast in current research

Although the importance of breakfast consumption to young children’s health is generally known, there is an increasing prevalence of children skipping breakfast [34, 35]. Food behaviors established in childhood are often continued into adulthood [36]. Therefore, it is necessary to identify the determinants of skipping breakfast. Based on these determinants, interventions for preventing skipping breakfast and promoting healthy dietary behaviors among children can be developed.

The parental role in the development of children’s healthy breakfast behaviors is not questioned [37]. Pearson et al. report in their review on family correlates of breakfast consumption that parental breakfast intake is associated with the breakfast intake of their children [38]. Furthermore, they found out that living in two-parent families also has a positive influence on children’s and adolescent’s breakfast consumption [38]. The research from Wendy & Campbell also shows that children with single parents are more likely to skip breakfast than those with two parents [39]. These findings confirm the results of the present study that also found evidence for a relation between family structures and skipping breakfast. Therefore, it is important to consider family structures of the children when designing programs to promote healthy breakfast behavior’s.

Associations between parenting and children’s breakfast consumption were found for permission to skip breakfast and parental self-efficacy of skipping breakfast which were negatively associated with children’s breakfast consumption [40]. Another study by Fugas et al. shows further reasons for skipping breakfast: lack of time, not being hungry in the morning and feeling unwell at the time of having breakfast are identified as explanations for skipping breakfast before going to school [41]. Similar to our findings that girls are more likely to skip breakfast than their male peers, other studies showed that breakfast consumption was more frequent among boys [42, 43]. This result could be explained by the likelihood that even young girls care more about their appearance and a slim figure [44]. A further study found out that adolescent boys and girls are more likely to skip breakfast if they perceived that their mothers often skip lunch [45]. Equal results are available for girls skipping breakfast with regard to their best friend’s meal skipping behaviors. On the contrary, those who reported exemplary maternal healthy eating behaviors were less likely to skip breakfast [45]. These findings underline the importance of interventions to address parents, children and their peers simultaneously.

The investigations of Keski-Rahkonen et al. and Utter et al. show an association between skipping breakfast and having a higher BMI [43, 46]. Research on breakfast intake and abdominal obesity parameters are rare. Alexander et al. report that eating breakfast was associated with lower visceral adiposity in overweight Latino youth, aged 10 to 17 years [11]. In a survey of Iranian children and adolescents aged 6 to 18 years the percentage of abdominal obesity in breakfast skippers was almost 5 percentage points higher than in non-skippers [47]. A French study in primary schoolchildren showed that those who regularly eat breakfast had the lowest waist circumferences [48].

There are many studies showing a positive relationship between skipping breakfast and a low socioeconomic status [42, 49]. In the present study, children with migration background were more likely to skip breakfast than their counterparts and a tertiary family education level was positively associated with having breakfast.

Children who spend more time in front of screen media and are less physically active are more likely to skip breakfast than their peers. These results are in line with the results of a study by Tin et al. in Hongkong with primary schoolchildren [49] and the study of Timlin et al. with adolescents [42]. Only one study found no relation between physical activity and skipping breakfast [43]. TV viewing during meals and the consumption of sugar sweetened beverages along with skipping breakfast were associated with significantly higher waist circumferences in French primary schoolchildren [48].

The probability of skipping breakfast increased with age [1]. The present study could not detect associations between age and skipping breakfast, probably because of the restricted variation in the age of the participating primary schoolchildren.

Last but not least, there should also be a focus on general meal patterns and obesity. Not just skipping breakfast is associated with obesity but a wide range of obesogenic behaviors influences weight status. Berg et al. showed an association between obesity and skipping breakfast, skipping lunch or eating at night. Larger self-reported portion size was also related to obesity. However, the investigation showed no significant relationship with intake of total energy [50]. Other authors emphasized that large, high energy-dense portions favor obesogenic eating behaviors in children [51]. Moreover, serving children large entrée portion sizes increases total energy intake but without decreases in intake of other foods. If children can self-select and limit their food intake, energy intake will decrease [52]. In this context, it seems necessary not only to consider breakfast skipping children when providing healthy breakfast at school. Attention should also be attached to children who had breakfast previously to avoid a higher intake of energy that might aggravate the situation and would be counterproductive in obesity prevention.

In summary, our study contributes to the body of evidence that exits for factors associated with skipping breakfast in primary schoolchildren. We identified vulnerable groups for targeted prevention and behavioral aspects, which need to be addressed in addition with preventing children from skipping breakfast. Therefore, the current results play an important role for developing targeted preventive measures for skipping breakfast in primary schoolchildren.

### Strengths and limitations

This research provides a valuable contribution in exploring determinants for the prevention of skipping breakfast in schoolchildren. However, some aspects should be considered when interpreting these findings.

Anthropometric measurements of the children were taken in a standardized manner according to a protocol by specially trained staff. Data management and statistical advice was provided by the Institute of Epidemiology and Medical Biometry at Ulm University. However, a limiting factor is the cross-sectional character of this research that precludes any causal interpretations of the results because cross-sectional studies do not allow conclusions about the direction of the detected associations.

Parents did not completely fill in questionnaires therefore missing values occurred. In observational studies, missing data are a frequently arising problem and may possibly bias the results. Therefore, the study examined the specific differences between those participants with missing and those with complete data. Considering these differences, children with missing data show several critical characteristics: They have higher rates of migration background and abdominal obesity, and less frequently a tertiary educational level. If these participants could have been included in the regression analysis for correlates of skipping breakfast, it might have accentuated the results. In this research, only schoolchildren whose teachers gave their agreement to participate were involved. Furthermore, due to the voluntary participation, only 62% of parents of eligible children gave their consent. Thus data from over a third of eligible children could not be collected. It may be assumed that there are differences between children who participated in the study and those who failed. This may contribute to the missing data bias.

In common with studies with an observational character, some unintentional bias may compromise the results. As already mentioned, only schoolchildren whose teachers and parents gave their agreement to participate were studied. Therefore, a twofold selection bias may have occurred on behalf of the teachers deciding to opt in and parents giving their consent for participation. Recall bias and social desirability bias may affect the parental report concerning the offspring’s patterns of physical activity, screen media use and consumption of soft drinks as well as breakfast habits. Parental breakfast was not assessed in the present study, but should be included in future research. Furthermore, the way skipping breakfast was assessed is a limitation of the study. Although the results are not representative for the whole of Germany, the sample size and the fact that this research comprises of data from an entire federal state of Germany are great strengths of the study. Response rates from participating parents with 87% at baseline were remarkably high. The study thus provides a valuable contribution for exploring determinants in the prevention of skipping breakfast in schoolchildren.

## Conclusion

Skipping breakfast contributes to the epidemic rise in childhood obesity. Therefore, it should be addressed in targeted interventions. Children, parents and teachers should be involved in those interventions preventing obesity and promoting health-conscious behavior and a healthy lifestyle. A special focus has to be given to girls, those who are already obese, have a migration background or a single parent and no tertiary family education level. Further crucial behaviors that are linked to breakfast habits as well as to obesity, and have to be addressed are physical activity, screen media use and consumption of soft drinks. Positive knowledge about breakfast consumption should be built up. Promoting and providing a healthy breakfast at school may particularly help breakfast skippers and should lie even in the interest of policymakers.

## Abbreviations

BMI: Body mass index
CASMIN: Comparative Analysis of Social Mobility in Industrial Nations
DRKS: German Clinical Trials Register
HOMA-IR: Homeostasis model assessment of insulin resistance index
ISAK: International Standards for Anthropometric Assessment
KiGGS: German Health Interview and Examination Survey for Children and Adolescents
NCD: Non-communicable disease
OR: Odds ratio
WC: Waist circumference
WHO: World Health Organization
WHtR: Waist-to-height ratio

## References

[1] Szajewska H, Ruszczyński M (2010). Systematic review demonstrating that breakfast consumption influences body weight outcomes in children and adolescents in europe. Crit Rev Food Sci Nutr.

[2] de la Hunty A, Gibson S, Ashwell M (2013). Does regular breakfast cereal consumption help children and adolescents stay slimmer? a systematic review and meta-analysis. Obes Facts.

[3] Baldinger N, Krebs A, Müller R, Aeberlie I (2012). Swiss children consuming breakfast regularly have better motor functional skills and are less overweight than breakfast skippers. J Am Coll Nutr.

[4] Wesnes KA, Pincock C, Scholey A (2012). Breakfast is associated with enhanced cognitive function in schoolchildren. An internet based study. Appetite.

[5] Donin AS, Nightingale CM, Owen CG, Rudnicka AR, Perkin MR, Jebb SA (2014). Regular breakfast consumption and type 2 diabetes risk markers in 9- to 10-year-old children in the child heart and health study in England (CHASE): a cross-sectional analysis. PLoS Med.

[6] Karatzi K, Moschonis G, Barouti A-A, Lionis C, Chrousos GP, Manios Y (2014). Dietary patterns and breakfast consumption in relation to insulin resistance in children. The Healthy Growth Study. Public Health Nutr.

[7] Freitas Júnior IF, Christofaro DGD, Codogno JS, Monteiro PA, Silveira LS, Fernandes RA (2012). The association between skipping breakfast and biochemical variables in sedentary obese children and adolescents. J Pediatr.

[8] Barr SI, DiFrancesco L, Fulgoni VL (2014). Breakfast consumption is positively associated with nutrient adequacy in Canadian children and adolescents. Br J Nutr.

[9] Kesztyüs D, Traub M, Lauer R, Kesztyüs T, Steinacker JM (2016). Correlates of longitudinal changes in the waist-to-height ratio of primary school children: Implications for prevention. Prev Med Reports.

[10] Alexander KE, Ventura EE, Spruijt-metz D, Weigensberg MJ, Goran MI, Davis JN (2010). Indices in Overweight Latino Youth. Obesity (Silver Spring).

[11] Hainer V, Aldhoon-Hainerova I (2013). Obesity paradox does exist. Diabetes Care.

[12] Bloom DE, Cafiero E, Jané-Llopis E, Abrahams-Gessel S, Reddy Bloom L, Fathima S, et al. The Global Economic Burden of Noncommunicable Diseases. In: World Economic Forum. 2011. w3.weforum.org/docs/WEF_Harvard_HE_GlobalEconomicBurdenNonCommunicableDiseases_2011.pdf. Accessed 8 Mar 2017.

[13] Coutinho T, Goel K, Corrêa De Sá D, Carter RE, Hodge DO, Kragelund C (2013). Combining body mass index with measures of central obesity in the assessment of mortality in subjects with coronary disease: Role of “normal weight central obesity. J Am Coll Cardiol.

[14] Javed A, Jumean M, Murad MH, Okorodudu D, Kumar S, Somers VK (2015). Diagnostic performance of body mass index to identify obesity as defined by body adiposity in children and adolescents: A systematic review and meta-analysis. Pediatr Obes.

[15] Griffiths C, Gately P, Marchant PR, Cooke CB (2013). A five year longitudinal study investigating the prevalence of childhood obesity: Comparison of BMI and waist circumference. Public Health.

[16] Kesztyüs D, Wirt T, Kobel S, Schreiber A, Kettner S, Dreyhaupt J (2013). Is central obesity associated with poorer health and health-related quality of life in primary school children? Cross-sectional results from the Baden-Württemberg Study. BMC Public Health.

[17] Rampersaud GC, Pereira MA, Girard BL, Adams J, Metzl JD (2005). Breakfast habits, nutritional status, body weight, and academic performance in children and adolescents. J Am Diet Assoc.

[18] Kesztyüs D, Schreiber A, Wirt T, Wiedom M, Dreyhaupt J, Brandstetter S (2013). Economic evaluation of URMEL-ICE, a school-based overweight prevention programme comprising metabolism, exercise and lifestyle intervention in children. Eur J Heal Econ.

[19] Dreyhaupt J, Koch B, Wirt T, Schreiber A, Brandstetter S, Kesztyues D (2012). Evaluation of a health promotion program in children: Study protocol and design of the cluster-randomized Baden-Wuerttemberg primary school study [DRKS-ID: DRKS00000494]. BMC Public Health.

[20] Brauns H, Steinmann S (1999). Educational reform in France, West-Germany and the United Kingdom: updating the CASMIN educational classification. ZUMA Nachrichten.

[21] Lange M, Kamtsiuris P, Lange C, Rosario AS, Stolzenberg H, Lampert T (2007). Messung soziodemographischer Merkmale im Kinder- und Jugendgesundheitssurvey (KiGGS) und ihre Bedeutung am Beispiel der Einschätzung des allgemeinen Gesundheitszustands [Sociodemographic characteristics in the German Health Interview and Examination Surve. Bundesgesundheitsblatt Gesundheitsforsch Gesundheitsschutz.

[22] Kurth B-M (2007). Der Kinder- und Jugendgesundheitssurvey (KiGGS): Ein Überblick über Planung, Durchführung und Ergebnisse unter Berücksichtigung von Aspekten eines Qualitätsmanagements TL - 50. Bundesgesundheitsblatt Gesundheitsforsch Gesundheitsschutz.

[23] World Health Organization. Global recommendations on physical activity for health. In: WHO Global Strategy on Diet, Physical Activity and Health. 2010. http://apps.who.int/iris/bitstream/10665/44399/1/9789241599979_eng.pdf Accessed 04 June 2016.

[24] Stewart A, Marfell-Jones M, Olds T, De Ridder H. International standards for anthropometric assessment. In: International Standards for Anthropometric Assessment. International Society for the Advancement of Kinanthropemetry. 2011. http://www.ceap.br/material/MAT17032011184632.pdf. Accessed 04 June 2016.

[25] Kromeyer-Hauschild K, Wabitsch M, Kunze D, Geller F, Geiß HC, Hesse V (2001). Perzentile für den Body-mass- Index für das Kindes- und Jugend- alter unter Heranziehung ver- schiedener deutscher Stichproben [Percentiles of bodymass index in children and adolescents evaluated from different regional German studies]. Monatsschrift Kinderheilkd.

[26] McCarthy HD, Ashwell M (2006). A study of central fatness using waist-to-height ratios in UK children and adolescents over two decades supports the simple message--‘keep your waist circumference to less than half your height’. Int J Obes.

[27] Nambiar S, Hughes I, Davies PS (2010). Developing waist-to-height ratio cut-offs to define overweight and obesity in children and adolescents. Public Health Nutr.

[28] World Health Organisation. Physical status: the use and interpretation of anthropometry. In: Report of a WHO Expert Committee. WHO Technical Report Series 854. 1995. http://apps.who.int/iris/bitstream/10665/37003/1/WHO_TRS_854.pdf. Accessed 04 June 2016. 8594834

[29] Ashwell M, Hsieh SD (2005). Six reasons why the waist-to-height ratio is a rapid and effective global indicator for health risks of obesity and how its use could simplify the international public health message on obesity. Int J Food Sci Nutr.

[30] Vandenbroucke JP, Von Elm E, Altman DG, Gøtzsche PC, Mulrow CD, Pocock SJ (2007). Strengthening the Reporting of Observational Studies in Epidemiology (STROBE): Explanation and elaboration. PLoS Med.

[31] Eldridge SM, Ukoumunne OC, Carlin JB (2009). The intra-cluster correlation coefficient in cluster randomized trials: a review of definitions. Int Stat Rev.

[32] Vasques C, Magalhães P, Cortinhas A, Mota P, Leitao J, Lopes VP (2014). Effects of intervention programs on child and adolescent BMI: a meta-analysis study. J Phys Act Health.

[33] Pereira MA, Erickson E, Mckee P, Schrankler K, Raatz SK, Lytle LA (2011). Breakfast frequency and quality May affect glycemia and appetite in adults and children 1–4. J Nutr.

[34] Shaw ME (1998). Adolescent breakfast skipping : an australian study. Adolescence.

[35] Nicklas TA, Bao W, Webber LS, Berenson GS (1993). Breakfast consumption affects adequacy of total daily intake in children. J Am Diet Assoc.

[36] Mikkilä V, Räsänen L, Raitakari OT, Pietinen P, Viikari J (2004). Longitudinal changes in diet from childhood into adulthood with respect to risk of cardiovascular diseases: the cardiovascular risk in young finns study. Eur J Clin Nutr.

[37] van der Horst K, Oenema A, Ferreira I, Wendel-Vos W, Giskes K, van Lenthe F (2007). A systematic review of environmental correlates of obesity-related dietary behaviors in youth. Health Educ Res.

[38] Pearson N, Biddle SJH, Gorely T (2009). Family correlates of breakfast consumption among children and adolescents. A systematic review. Appetite.

[39] Wolfe WS, Campbell CC (1993). Food pattern, diet quality, and related characteristics of schoolchildren in New York State. J Am Diet Assoc.

[40] Van Lippevelde W, Te Velde SJ, Verloigne M, Van Stralen MM, De Bourdeaudhuij I, Manios Y (2013). Associations between family-related factors, breakfast consumption and BMI among 10- to 12-year-Old european children: the cross-sectional ENERGY-study. PLoS One.

[41] Fugas V, Berta E, Walz F, Fortino A, Martinelli MJ (2013). Breakfast habit and quality in students from two public primary schools in the city of Santa Fe. Arch Argent Pediatr.

[42] Timlin MT, Pereira MA, Story M, Neumark-Sztainer D (2008). Breakfast eating and weight change in a 5-year prospective analysis of adolescents: project EAT. Pediatrics.

[43] Utter J, Scraagg R, Mhurchu CN, Schaaf D (2007). At-home breakfast consumption among New index and related nutrition behaviors. J Am Diet Assoc.

[44] Ricciardelli LA, McCabe MP (2001). Children’s body image concerns and eating disturbance: a review of the literature. Clin Psychol Rev.

[45] Pearson N, Williams L, Crawford D, Ball K (2012). Maternal and best friends’ influences on meal-skipping behaviours. Br J Nutr.

[46] Keski-Rahkonen A, Kaprio J, Rissanen A, Virkkunen M, Rose RJ (2003). Breakfast skipping and health-compromising behaviors in adolescents and adults. Eur J Clin Nutr.

[47] Ahadi Z, Qorbani M, Kelishadi R, Ardalan G, Motlagh ME, Asayesh H (2015). Association between breakfast intake with anthropometric measurements, blood pressure and food consumption behaviors among Iranian children and adolescents: the CASPIAN-IV study. Public Health.

[48] Isacco L, Lazaar N, Ratel S, Thivel D, Aucouturier J, Doré E (2010). The impact of eating habits on anthropometric characteristics in French primary school children. Child Care Health Dev.

[49] Tin S, Ho S, Mak K, Wan K, Lam T (2011). Lifestyle and socioeconomic correlates of breakfast skipping in Hong Kong primary 4 schoolchildren. Prev Med (Baltim).

[50] Berg C, Lappas G, Wolk A, Strandhagen E, Toren K, Rosengren A, Thelle D, Lissner L (2009). Eating patterns and portion size associated with obesity in a Swedish population. Appetite.

[51] Fisher JO, Kral TV (2008). Super-size me: Portion size effects on young children’s eating. Physiol Behav.

[52] Fisher JO, Rolls BJ, Birch LL (2003). Children’s bite size and intake of an entrée are greater with large portions than with age-appropriate or self-selected portions. Am J Clin Nutr.

